# Fat Grafting With Harvesting From Zone IV in the DIEP Flap

**Published:** 2019-05-31

**Authors:** Yoshihiro Sowa, Takuya Kodama, Daiki Morita, Toshiaki Numajiri

**Affiliations:** Departments of Plastic and Reconstructive Surgery, Graduate School of Medical Sciences, Kyoto Prefectural University of Medicine, Kyoto, Japan

**Keywords:** deep inferior epigastric artery perforator, fat grafting, breast reconstruction, breast augmentation, breast cancer

## CASE DESCRIPTION

**Figure 1:**
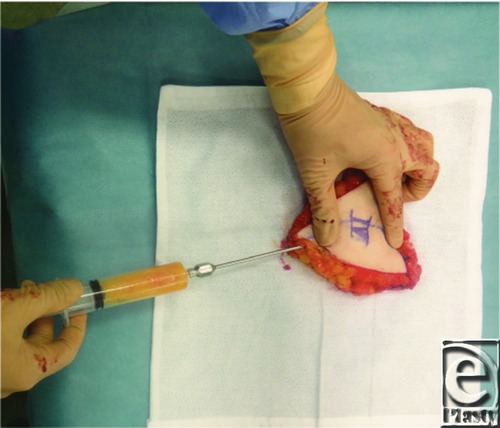
Intraoperative image showing fat tissue suction from zone IV in the DIEP flap using the wet technique with a 3-mm cannula and a 20-mL Luer-Lok syringe. DIEP indicates deep inferior epigastric artery perforator.

**Figure 2:**
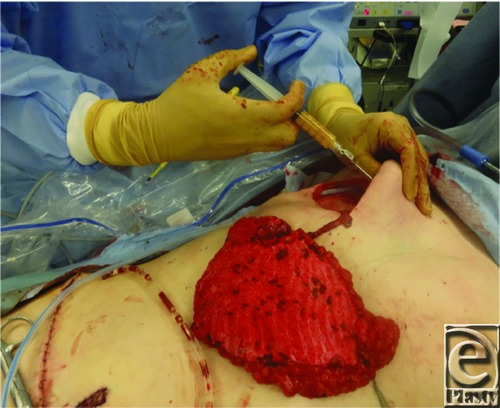
Intraoperative image showing additional fat grafting in the pectoralis major muscle.

**Figure 3:**
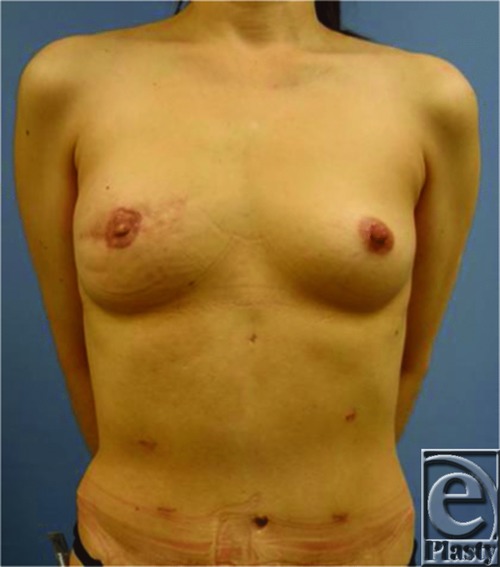
Postoperative image of the patient 1 year after left breast skin–sparing mastectomy and DIEP flap reconstruction combined with fat grafting with harvesting from zone IV in the DIEP flap. DIEP indicates deep inferior epigastric artery perforator.

The patient was a 43-year-old woman with invasive ductal carcinoma in the left breast who was treated with skin-sparing mastectomy. She then underwent total breast reconstruction using a deep inferior epigastric artery perforator (DIEP) flap simultaneously with fat grafting with harvesting from zone IV in the DIEP flap. In the procedure, fat tissue was harvested from zone IV in the DIEP flap using the wet technique with a 3-mm cannula and a 20-mL Luer-Lok syringe under manually generated negative pressure (Fig 1). Fat was centrifuged at 2000 rpm for 2 minutes and then injected with a blunt Coleman cannula and 5-mL syringes. Fat injections were performed along the muscular fascicle of the pectoralis major in the subcutaneous tissue, if possible into the subcutaneous layer (Fig 2). The volume of the fat graft depends on the size of the harvested DIEP flap, but 20 to 30 mL of fat tissue can usually be injected. In this case, there was no local recurrence or systemic metastasis during a 2-year follow-up period, and no cysts were detected by ultrasonography, indicating that no fat necrosis occurred. The patient was highly satisfied with the cosmetic results (Fig 3).

## QUESTIONS

1. Describe zone IV in the Hartrampf perfusion zones of the lower abdominal flap.
2. How can zone IV in the DIEP flap be used effectively?
3. How is fat harvested from zone IV in the DIEP flap?
4. What are the advantages and disadvantages of the fat grafting procedure?

## DISCUSSION

The DIEP flap has become an increasingly common autologous reconstructive choice after mastectomy due to its volume, good color and texture, low donor site morbidity, and success rates comparable with those for other flaps.^1^ However, compared with other autologous flaps, the DIEP flap has disadvantages of greater technical difficulty using all parts of the flap and a greater incidence of partial fat necrosis that affects the cosmetic outcome and the patient's satisfaction.^2^ The Hartrampf perfusion zones of the lower abdominal flap divide the abdominal ellipse into 4 equal parts (zones I, II, III, and IV) with different perfusion. Among these parts, zone IV is considered to be the most unstable because it is located most distant from zone I, which includes perforator vessels. Therefore, zone IV is routinely discarded to avoid risks of partial flap loss and fat necrosis.^2^^,^^3^ However, it is not certain whether this zone has to be discarded if augmentation is not used.

The natural slope from the low neckline to the upper pole of the breast, which is called the décolleté line, is sometimes reconstructed by broadly paving the flat distal part of zone III of the DIEP flap. However, we often find that this part cannot be used because of blood flow insufficiency, unless bilateral perforators are included in the DIEP flap. In contrast, the central part of zone III has relatively stable blood flow but is too thick to be used for augmentation of the décolleté line on the upper chest. Fat grafting is helpful in implant-based breast reconstruction for augmenting the breast volume, improving contour irregularities, and optimizing aesthetic results.^4^

Recently, a few reports have also shown the value of fat grafting in autologous reconstruction.^5^^-^7 In the DIEP flap, zone IV is a suitable donor site for fat tissue for use in grafting for augmentation of dents or step-offs in the area around the décolleté line. In our cases, we actually inject fat tissue into the pectoralis major muscle and around tissue including the subcutaneous layer.

Breast reconstruction combined with fat grafting is effective for correction of deformities, especially in the décolleté region after breast reconstruction with an abdominal flap.^7^ The advantages of this procedure include simple and rapid application, low cost, minimal scarring, and efficacy for simultaneous restoration of multiple irregular defects. Common disadvantage of injectable soft tissue fillers is their limited longevity and potential formation of cysts caused by fat necrosis.^8^ However, recent technological advances have made autologous fat grafting an effective method for restoring soft tissue defects.^4^

## SUMMARY

Fat grafting with harvesting from zone IV in a DIEP flap is an ideal option for cosmetic breast augmentation in patients who wish to achieve moderate, natural enlargement of the décolleté line on the upper chest.
